# Leisure‐time physical activity predicts levels of advanced glycation end‐products in older women: A 15‐year follow up from the Helsinki Birth Cohort Study

**DOI:** 10.1111/ggi.70049

**Published:** 2025-05-01

**Authors:** Mathias Lundström, Niko Wasenius, Mia Eriksson, Tuija M Mikkola, Eero Kajantie, Johan G Eriksson

**Affiliations:** ^1^ Folkhälsan Research Center Helsinki Finland; ^2^ Doctoral Programme of Population Health, Faculty of Medicine, University of Helsinki Helsinki Finland; ^3^ Department of General Practice and Primary Health Care University of Helsinki and Helsinki University Hospital Helsinki Finland; ^4^ Clinicum, Faculty of Medicine University of Helsinki Helsinki Finland; ^5^ Population Health Unit Finnish Institute for Health and Welfare Helsinki Finland; ^6^ Clinical Medicine Research Unit, MRC Oulu University of Oulu and Oulu University Hospital Oulu Finland; ^7^ Department of Clinical and Molecular Medicine Norwegian University of Science and Technology Trondheim Norway; ^8^ Singapore Institute for Clinical Sciences (SICS) Agency for Science, Technology and Research (A*STAR) Singapore Singapore; ^9^ Department of Obstetrics and Gynecology and Human Potential Translational Research Program, Yong Loo Lin School of Medicine National University of Singapore Singapore Singapore

**Keywords:** advanced glycation end‐products, cohort study, follow‐up study, old age, physical activity

## Abstract

**Aim:**

Physical activity might be able to delay the aging process by reducing levels of advanced glycation end‐products (AGEs). However, the influence of physical activity on levels of AGEs remains unclear. We investigated the associations between leisure time physical activity (LTPA) in late midlife and change in LTPA during a 15‐year follow up on the levels of AGEs in old age.

**Methods:**

We analyzed 767 participants from the Helsinki Birth Cohort Study. LTPA was measured with a validated questionnaire in late midlife and in old age. The levels of AGEs were measured by skin autofluorescence in old age. General linear models and restricted cubic regression spline models were used to study the associations between LTPA and AGEs. Analyses were adjusted for age, alcohol consumption, dietary index, smoking, follow‐up time, body mass index and socioeconomic status.

**Results:**

Mean levels of AGEs in women (2.33 AU, SD 0.46) were lower than in men (2.49 AU, SD 0.50, *P* < 0.001). Women in the lowest LTPA quartile had 0.19 AU (95% CI 0.07–0.32, *P* = 0.002), 0.21 AU (95% CI 0.09–0.33, *P* = 0.001) and 0.18 AU (95% CI 0.05–0.31, *P* = 0.006) higher levels of AGEs compared with women in the second, third and fourth quartile. In the restricted cubic regression spline model, levels of AGEs (*P* = 0.006) were decreasing with increasing LTPA from 0 to 32 METh/week, after which the association plateaued. No associations were found in men.

**Conclusions:**

Greater volume of LTPA in late midlife is associated with lower levels of AGEs in skin tissue in old age in women. **Geriatr Gerontol Int 2025; 25: 781–788**.

## Graphical abstract

This longitudinal study with 767 participants from the Helsinki Birth Cohort Study found that greater volume of leisure time physical activity in late midlife is associated with lower levels of advanced glycation end products in old age in women.

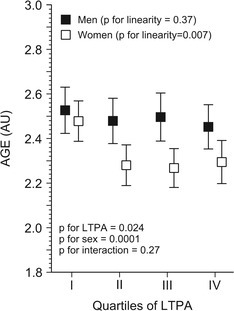

## Introduction

In recent decades, lifespan has been increasing faster than health span.^1^ Aging is associated with increasing risk of chronic diseases, and preventative measures are subsequently needed to extend health span.^2^ Physical activity (PA) is an accessible measure to prevent chronic disease and frailty in old age.^3^

Advanced glycation end‐products (AGEs) are proteins and lipids that become glycated in the presence of sugars through the Maillard reaction.^4^ AGEs found in human tissues increase with age, smoking, consumption of foods cooked at high‐temperatures and high levels of simple carbohydrates.^5^ AGEs are associated with oxidative stress and systemic inflammation^6^ through mechanisms, such as binding to the major receptor for AGEs (RAGE) with a downstream effect of inducing vascular damage.^7^ Targeting the AGE–RAGE axis has been proposed as a novel therapy for AGE‐related diseases.^8^ AGEs have been proposed as a marker for aging,^9^ and have been associated with chronic diseases, such as cardiovascular disease, neurodegenerative diseases and depression.^10^, ^11^, ^12^ AGEs in skin emit fluorescence, and can be non‐invasively measured with a skin autofluorescence (SAF) reader.^13^ SAF has been shown to be associated with lifestyle factors and diabetic complications, making it a potential tool of risk assessment of AGE‐related diseases.^14^

PA can protect against deleterious effects of AGEs by improving metabolic status.^15^ In individuals with diabetes, PA is associated with improved glycemic control and subsequent formation of AGEs.^16^ However, studies addressing the associations between PA and AGEs have reported conflicting results. Couppé *et al*. observed an association between lifelong endurance running and lower levels of AGEs in connective tissue in physically active men.^17^ In another study on healthy individuals from Slovakia, regular PA was found to be associated with lower levels of AGEs, as measured by SAF.^18^ Drenth *et al*. reported that in the older population, higher levels of AGEs are associated with lower levels of PA and physical performance, discussing the possible bidirectional relationship between PA and AGEs.^19^ Conversely, in a study of Dutch adults, no association between moderate to vigorous PA and AGEs was found, although an association was found in the chronic diseases group.^20^ Another study focusing on middle aged individuals without known cardiovascular disease or diabetes also found no association between PA and AGEs.^21^

Our aim is to analyze whether the volume of leisure‐time physical activity (LTPA) in late midlife, and the change in the volume of LTPA in late midlife to old age, during a 15‐year follow‐up period in the participants of the Helsinki Birth Cohort Study, can predict levels of AGEs in old age. We hypothesize that higher levels of LTPA in late midlife would be associated with lower levels of AGEs in old age, and that increasing or maintaining higher levels of LTPA during the 15‐year follow‐up period would be associated with lower levels of AGEs in old age.

## Methods

### Study population

For this longitudinal study with a 16‐year follow‐up time, where LTPA was measured at late midlife, change in LTPA from late midlife to old age and AGEs at old age, we applied data from the Helsinki Birth Cohort Study. The Helsinki Birth Cohort Study is a cohort of originally 13 345 men and women born between 1934 and 1944 in one of two public maternity hospitals in Helsinki, Finland. A total of 8760 individuals born at Helsinki University Hospital form the baseline cohort of the present study. The cohort members attended child welfare clinics, and the majority attended school in the Helsinki region. By 1971, all Finnish citizens and permanent residents had received a unique identification number. In 2001–2004 (late midlife) 2902 individuals were randomly selected, out of which 2003 individuals participated in a clinical examination. In 2011–2013, 1404 of the prior participants were still alive and living within 100 km of the study clinic in Helsinki; of these 1404 invited participants, 1094 individuals participated. Finally, in 2017–2018 (old age) participants who were still alive and living within 100 km of the study clinic in Helsinki were invited to participate in further studies. Of those invited, 815 individuals participated in the clinical follow up. Excluding 48 participants because of missing data, our final sample comprised 767 participants (*n* = 339 men and *n* = 428 women) who had complete data on AGEs in 2017–2018, the main predictor LTPA, and covariates age, alcohol consumption, dietary score, smoking, follow‐up time, body mass index and maximum socioeconomic status in adulthood (Fig. S3).

### Ethics

The Coordinating Ethical Committee of the Hospital District of Helsinki and Uusimaa approved the study. All participants gave their written informed consent before participation.

### LTPA

LTPA was measured in late midlife (mean age 61 years) and old age (mean age 75 years) using the validated physical activity questionnaire Kuopio Ischemic Heart Disease questionnaire. The Kuopio Ischemic Heart Disease questionnaire assesses the participants' history of LTPA in the past 12 months.^22^ The Kuopio Ischemic Heart Disease questionnaire accounts for conditioning PA (e.g. running, skiing, swimming), non‐conditioning LTPA (e.g. gardening, housework, snow shoveling), PA from commuting to work (e.g. walking, cycling) and, in addition, a category of “other” PA specified by the participant. Frequency, duration and intensity (0 = no sweating/no shortness of breath, 1 = no sweating/shortness of breath, 2 = sweating/no shortness of breath, 3 = sweating/shortness of breath) of the PA is reported for each activity. For each mode of activity and its intensity category, a metabolic equivalent of task (MET) value was assigned based on previously published databases.^23^ MET values describe the ratio of metabolic rate, specifically oxygen consumption, during an activity compared with metabolic rate at rest. Details of the calculations have been published previously.^24^ We calculated MET‐hours (METh) per week, which describe the total volume of LTPA by adding the products of MET × frequency × duration for each activity. The total volume of LTPA was categorized into sex‐specific quartiles (in men: I <21.5 METh/week, II ≥21.5 to <36.0 METh/week, III ≥36.0 to <59.7 METh/week and IV ≥59.7 METh/week, and in women: I <21.5 METh/week, II ≥21.5 to <35.7 METh/week, III ≥35.7 to <59.0 METh/week and IV ≥59.0 METh/week). LTPA was also used as a continuous variable in the statistical analyses. LTPA was measured similarly in late midlife (2001–2004) and during follow up in old age (2017–2018).

### AGE levels

Levels of AGEs were measured as SAF using an AGE reader (214B00102; DiagnOptics, Groningen, the Netherlands) on participants' dominant forearm. SAF is expressed as arbitrary units (AU), representing the ratio of emitted light intensity from the AGE reader (420–600 nm wavelength range) and the reflected excitation light intensity from the skin (300–420 nm wavelength range) multiplied by 100.^13^

### Diet

Participants' dietary habits were assessed using a validated food frequency questionnaire measuring the habitual diet over the past 12 months.^25^, ^26^ The average consumption frequency and portion size of each food frequency questionnaire item was registered by the participants at the study site under the supervision of a trained study nurse. Daily food items, and energy and nutrient intake were calculated using the Finnish National Food Composition Database (Fineli).^27^

The dietary index was calculated based on the 2005 Finnish dietary recommendations^28^ and accounts for eight variables, including four food groups (fruits, vegetables, the ratio of white to red meat and the consumption of rye representing fiber intake) and four nutrient groups (fatty acids, alcohol, salt and sucrose). The level of consumption of each component was categorized into quartiles and assigned a score between 0 and 3. The total score ranged from 0 to 24 AU. The recommended Finnish diet score has been previously described in detail by Kanerva *et al*.^29^

### Covariates

A 75‐g 2‐h oral glucose tolerance test was carried out with measurements of glucose and insulin. Fasting glucose, insulin and serum lipids were measured with standard laboratory analyses from blood drawn after an overnight fast. Height was measured using a Kawi stadiometer, and weight was measured with a Seca Alpha 770 scale (Hamburg, Germany). Body mass index (BMI) was calculated as kg/m^2^. Blood pressure was measured with a sphygmomanometer from the participants right arm in a seated position and the mean value from two successive readings was recorded. Information on smoking habits, alcohol consumption and socioeconomic status was gathered through questionnaires.

### Statistical analysis

Means and standard deviations are reported for continuous variables, and frequencies and percentages for categorical variables. Analyses of variance for continuous variables, and the χ^2^‐test for categorical variables were used to test differences in baseline characteristics between LTPA quartiles. For the categorical variables, linear regression analyses were carried out to study associations between categorical volume of LTPA in late midlife (mean age of 61 years) and AGEs in old age (mean age of 76 years). We included the interaction term between sex and LTPA categories in the model to assess sex differences. For the continuous variables, restricted cubic spline model with four knots and bootstrap‐based confidence intervals (10 000 repetitions) was used separately for men and women to test the associations between continuous LTPA in late midlife and AGE in old age. We also tested the association between standardized residual change in LTPA from late midlife to old age, and levels of AGEs in old age. Both continuous and categorized (≤−0.5 SD, >−0.5 to <0.5 SD, ≥0.5 SD) standardized residual change in LTPA were used. Sex‐specific residual change scores were calculated by first predicting LTPA in old age with LTPA in late midlife, then the predicted values were subtracted from the volume of LTPA in old age. Due to the high correlation between the change in LTPA from late midlife to old age and LTPA in midlife, the residual change scores were used. The models with change in LTPA as a dependent variable included sex by LTPA interaction term.

Both crude and fully adjusted models were analyzed. The crude models were adjusted for age and follow‐up time, and the fully adjusted models were adjusted for age, alcohol consumption, dietary index, smoking, follow‐up time, BMI and maximum social class in adulthood. We furthermore used linear regression analysis to examine the associations between covariates and AGEs with one minimally adjusted model with adjustment for age and follow‐up time, and one fully adjusted model with adjustment for age, follow‐up time, maximum social class, smoking and alcohol consumption. All covariates were measured in late midlife, and AGEs were measured in old age.

The threshold for statistical significance was set to *P* < 0.05. All analyses were carried out with Stata/MP 17.0 (StataCorp, College Station, TX, USA).

## Results

### Characteristics of participants in late midlife

Tables S1 and S2 show characteristics of men (*n* = 339) and women (*n* = 428) stratified by LTPA quartile, *P*‐values are for trend. Men in the highest LTPA quartile were older (*P* = 0.0005), and they had lower 30‐min (*P* = 0.01) and 2‐h (*P* = 0.0006) glucose concentrations than men in the lowest quartile. Women in the highest LTPA quartile were older compared with the lowest quartile (*P* = 0.04).

### Sex differences in LTPA and AGE

The mean volume of LTPA in late midlife was 46.3 METh/week (SD 34.9) in men and 43.9 METh/week (SD 35.5) in women. No significant differences were found after adjusting for age in late midlife (*P* = 0.34) or further adjusting for smoking, alcohol consumption, dietary index, body mass index and maximum socioeconomic status in adulthood (*P* = 0.84). AGE levels, however, were lower in women (mean 2.33 AU, SD 0.46) than in men (mean 2.49 AU, SD 0.50). The mean difference in AGE levels was 0.16 (95% CI 0.09–0.22, *P* < 0.0001) in the crude model and 0.16 AU (95% CI 0.08–0.24, *P* = 0.0001) in the fully adjusted model.

### LTPA in late midlife and AGEs in old age

**Figure 1:**
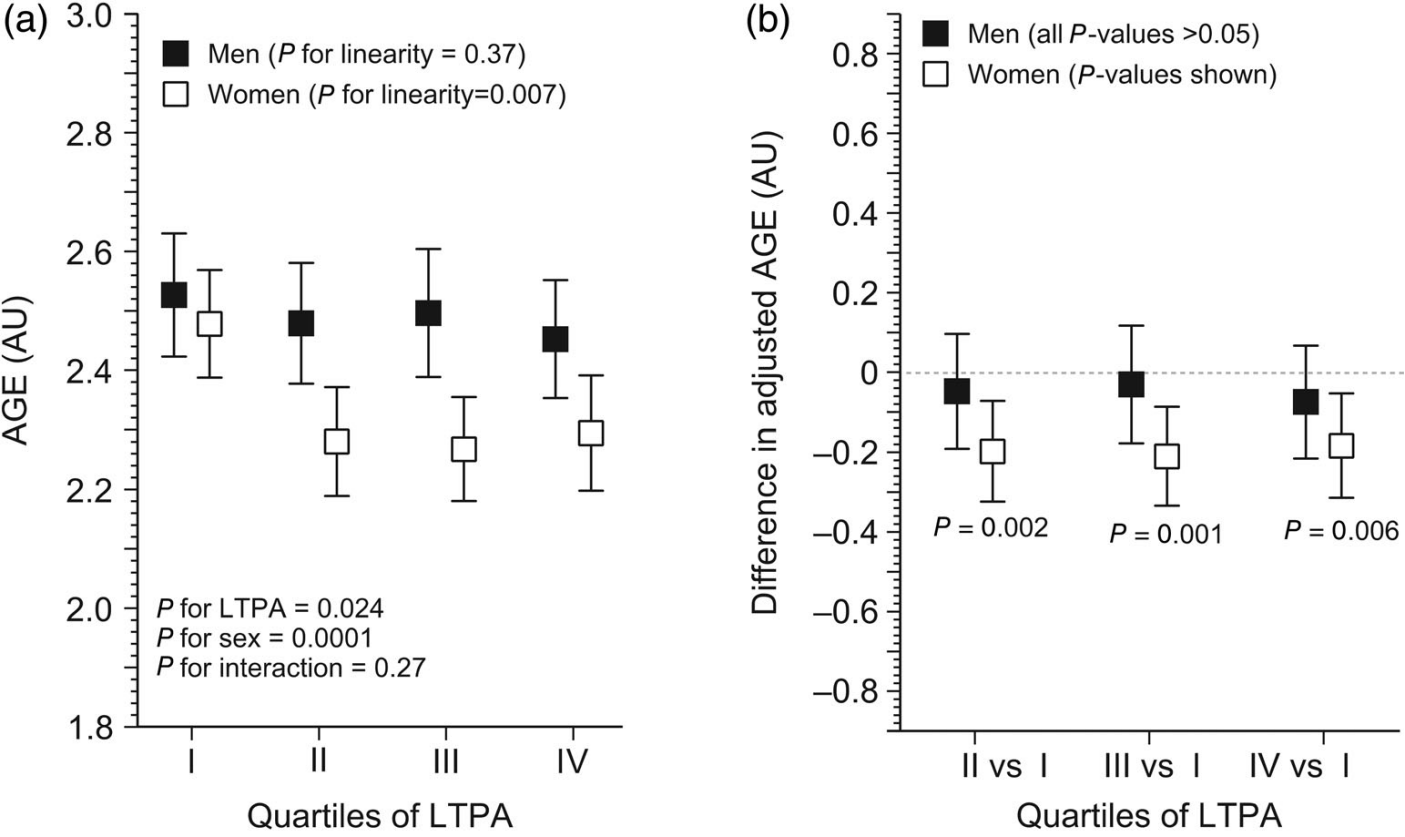
Fully adjusted association between the volume of leisure‐time physical activity (LTPA) quartiles in late midlife and advanced glycation end‐products (AGEs) in men and women in old age. AU, arbitrary units.

**Figure 2:**
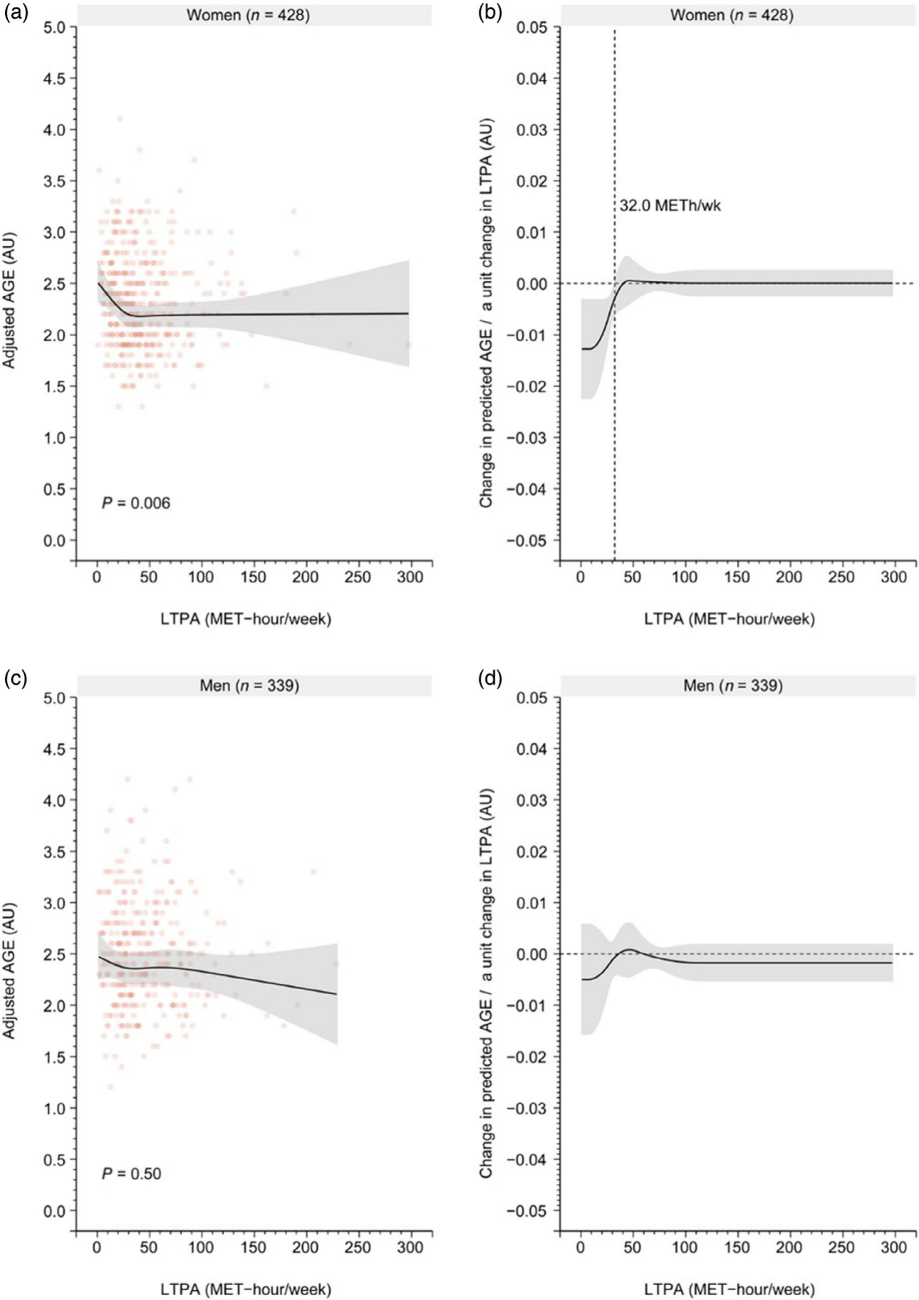
Fully adjusted predictions and marginal effect of restricted cubic spline regression models between the leisure‐time physical activity (LTPA) in late midlife and advanced glycation end‐products (AGEs) in men and women in old age. AU, arbitrary units; BMI, body mass index; MET, metabolic equivalent task.

We found a significant negative association between LTPA categories in late midlife and AGE levels in old age both in the crude (*P* = 0.024) (Fig. S1) and fully adjusted model (*P* = 0.024; Fig. 1). No significant interaction was found between men and women in this association, neither in the crude (*P* = 0.26) nor fully adjusted model (*P* = 0.27). However, the associations appear to be stronger in women than in men, as shown in Fig. 1. We found a significant association between continuous LTPA and late midlife and AGE values in old age in women in both the crude (*P* = 0.0046) (Fig. S2) and fully adjusted model (*P* = 0.006; Fig. 1), which was not the case for men. In women, AGE levels decreased rapidly between 0 and 32 METh/week, after which the association plateaued (Fig. 2).

### Change in LTPA from late midlife to old age, and association with AGEs

**Figure 3:**
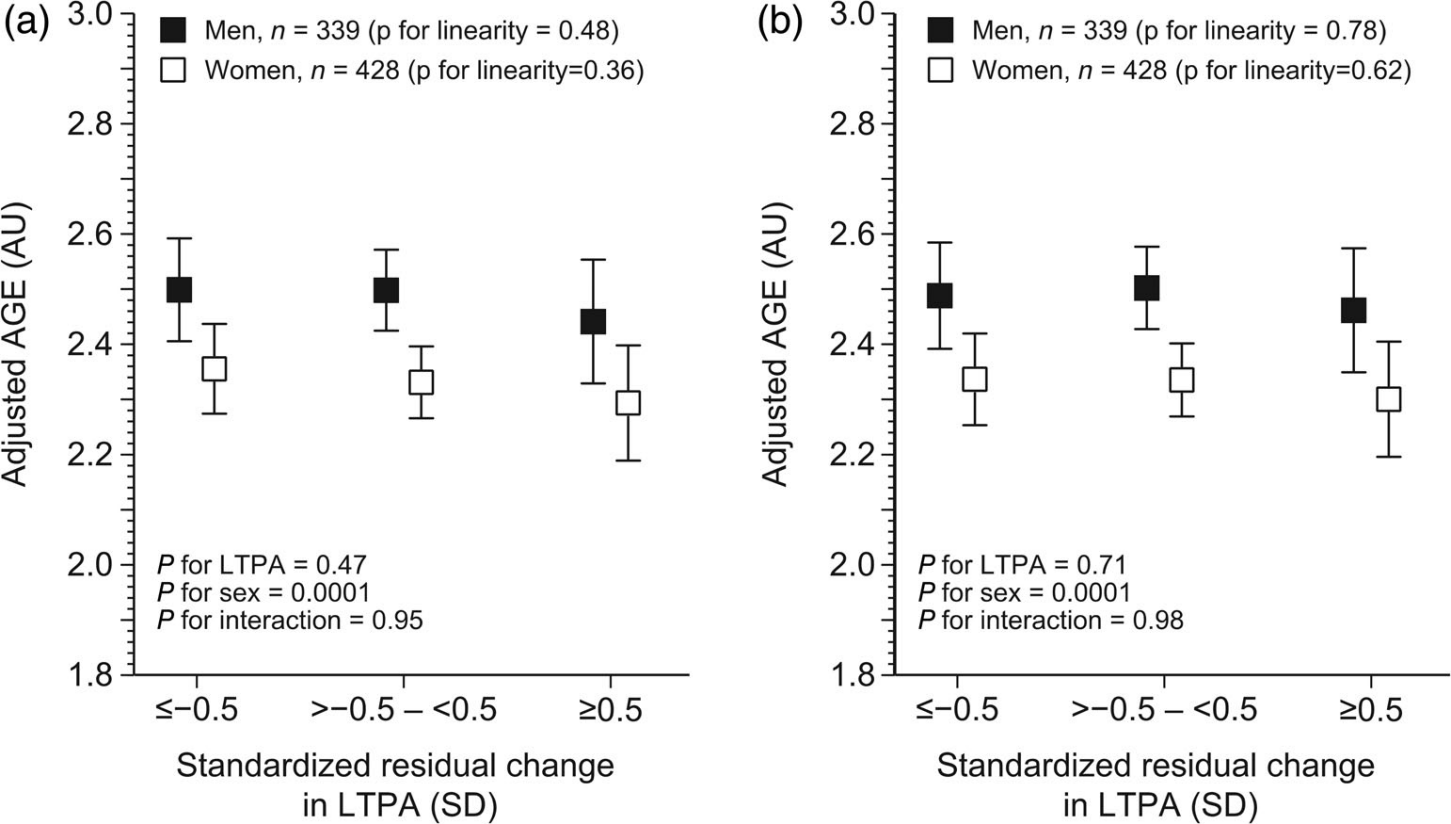
The (a) minimally and (b) fully adjusted association between the standardized residual change in categorical leisure‐time physical activity from late midlife into old age and advanced glycation end‐products (AGE) in old age. AU, arbitrary units; BMI, body mass index; LTPA, leisure‐time physical activity; MET, metabolic equivalent task; SD, standard deviation.

**Figure 4:**
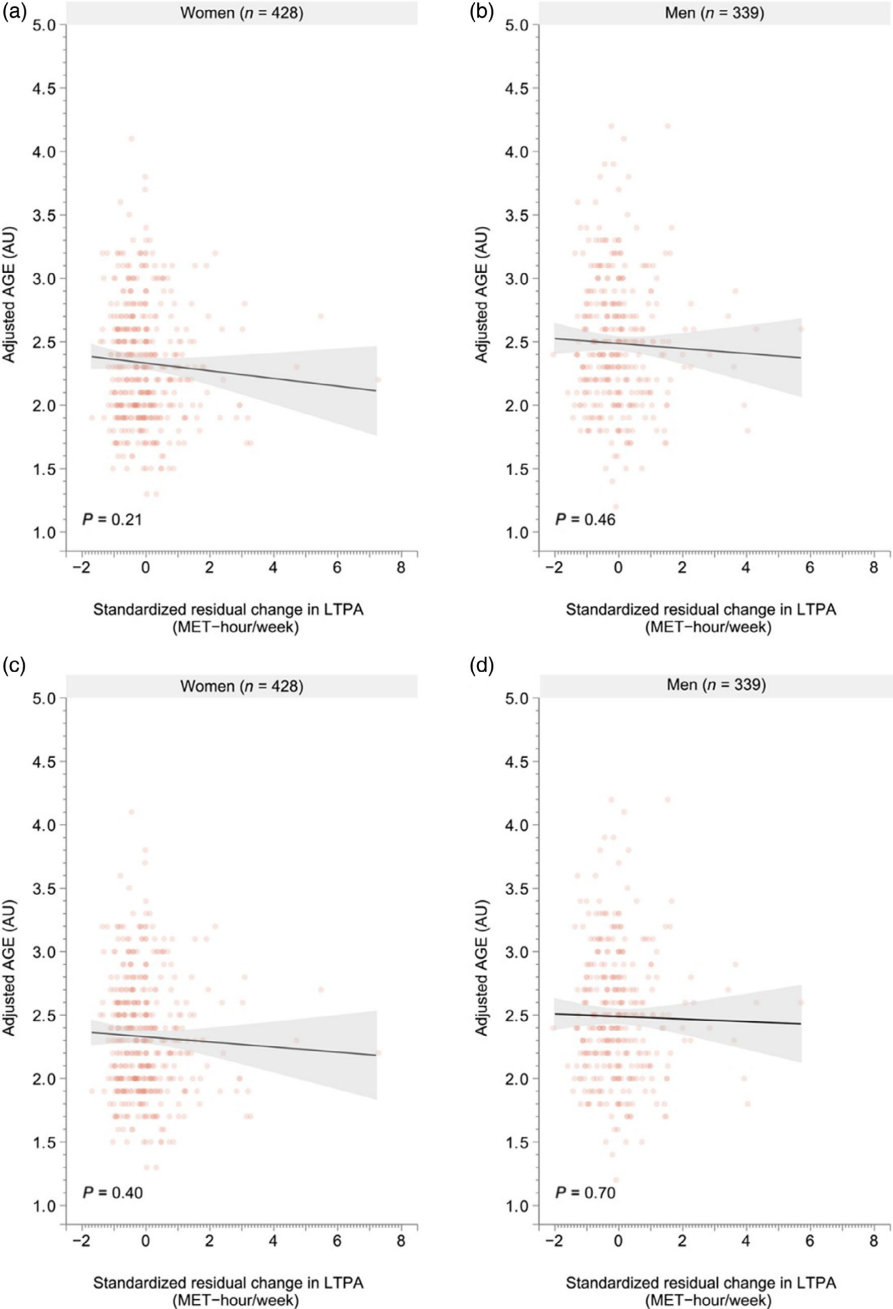
Minimally (a, b) and fully adjusted (c, d) association between the standardized residual change in continuous leisure‐time physical activity from late midlife into old age and AGE at old age. AU, arbitrary units; BMI, body mass index; LTPA, leisure‐time physical activity; MET, metabolic equivalent task; SD, standard deviation.

No significant associations were found between the level of AGEs and categorized or continual residual change in the volume of LTPA (from late midlife to old age) in either men or women. Similar findings were obtained in both the crude and fully adjusted models, as shown in Figures 3, 4. We found no significant interaction effect between continuous standardized residual change of LTPA and sex on AGE levels (*P* for interaction = 0.77). No significant association between the continuous standardized residual change in the volume of LTPA was found in either men (crude model: −0.02 AU, 95% CI −0.07–0.03, *P* = 0.46, and fully adjusted mode: −0.01 AU, 95% CI −0.06–0.04, *P* = 0.70) or women (crude model: −0.03 AU, 95% CI −0.08–0.01, *P* = 0.21, and fully adjusted model: −0.02 AU, 95% CI −0.07–0.03, *P* = 0.40).

### Association between covariates in late midlife and AGEs in old age

In the fully adjusted model in men (Table S3) after adjusting for age, follow up time, maximum social class in adulthood, smoking and alcohol consumption, significant associations were found for BMI (*b* = 0.022, 95% CI 0.007–0.037, *P* = 0.003), glucose levels at fasting (*b* = 0.119, 95% CI 0.063–0.176, *P* = 0.00004), glucose levels after 30 min (*b* = 0.048, 95% CI 0.022–0.075, *P* = 0.0004), glucose levels after 120 min (*b* = 0.024, 95% CI 0.007–0.041, *P* = 0.007) and fasting insulin (*b* = 0.012, 95% CI 0.006–0.019, *P* = 0.0002). In the minimally adjusted model adjusted for age and follow‐up time, there was a significant association for high‐density lipoprotein that lost significance in the fully adjusted model, otherwise the results were similar. No significant associations between covariates in late midlife and AGEs in old age were found in women (Table S4).

## Discussion

In the present study, we observed that volume of LTPA in late midlife was associated with levels of AGEs measured 15 years later in women. This was not the case for men. The low‐volume LTPA group had significantly higher levels of AGEs, and the threshold for significance was found to be approximately 32 METh/week, after which the effect plateaus. We found no significant association between the change in LTPA from late midlife to old age and the levels of AGEs.

Reducing sedentary behavior has been associated with a healthier aging trajectory.^30^ The threshold we observed at 32 METh/week corresponds to 80 min of brisk walking per day. With a higher volume of LTPA, the association with levels of AGEs lost significance.

SAF has been reported to increase linearly with age,^31^ but in the present study, both age and follow‐up time were included in the analyses, adjusting for the possibility that levels of AGEs would be observed as a function of age. Prolonged periods of exercise with insufficient recovery time have been associated with higher levels of inflammation,^32^ possibly contributing to the observed threshold. No significant effect was found on the association between change in LTPA over time and levels of AGEs. The lack of baseline data on AGE‐levels in the present study might contribute to this observation. The fact that PA levels are expected to decrease with increasing age^3^ and that in the present study we analyzed the total volume of PA rather than the relative intensity is furthermore a possible factor influencing the null result. In the present study, the individual LTPA levels were robust with increasing age, which might explain the association with baseline LTPA and the lack of a significant association with change in LTPA.

In the present study, there was an association between LTPA and AGEs in women, but not in men. In a longitudinal cohort, Kilhovd *et al*. showed that AGEs measured by serum immunoassays were associated with increased mortality related to coronary heart disease in nondiabetic women, but not in men.^33^ In contrast, Whitson *et al*. found a cross‐sectional relationship between AGEs measured by serum carboxymethyl‐lysine levels and frailty status only in men, discussing the possibility of sex‐specific survivor bias in aged cohorts.^34^ A publication by Koetsier *et al*. on SAF reference values showed no baseline differences between sexes, but women who smoked had higher AGE levels than men who smoked, suggesting that lifestyle factors could have a stronger impact on AGE levels in the female population.^31^

When analyzing the associations between covariates in late midlife and AGE levels in old age, we found significant associations for BMI, glucose levels and fasting insulin in men, but not in women. Sex hormones have been shown to influence insulin sensitivity and risk of metabolic syndrome,^35^ which could impact AGE levels. Sex hormones have also been shown to affect the rate of collagen turnover^36^ in skin, which could impact SAF levels. However, our study population was aged between 60 and 85 years, and it can be assumed that menopause has already occurred in women, thus decreasing the impact of estrogen on our findings. A study by Ebert *et al*. found that higher levels of AGEs were associated with lower physical functioning in women, but not in men, and they found no significant difference between age groups.^37^ Taken together, these results would suggest sex differences in AGE metabolism, but further interpretations from our study would be speculative.

The present results add to the discussion about the relationship between PA and AGEs in previous studies.^17^, ^18^, ^19^, ^20^, ^21^ Comparison between the studies is, however, difficult, due to the diversity in applied measures of PA and AGEs. The level of PA is often measured by self‐reports, rendering a source of subjectivity, and different patterns of PA can have different physiological effects. AGEs measured in skin tissue are thought to accumulate over several years compared with AGEs measured in serum, influenced by more recent changes in metabolism and oxidative stress.^38^ Furthermore, the causality between lifestyle factors, natural aging and AGEs is difficult to establish due to the co‐existence of the variables.^7^ However, knowing that PA improves glycemic control,^39^ contributes to redox homeostasis^40^ and lowers inflammation,^41^ it is plausible that PA would decrease the rate of AGE accumulation measured by SAF.

The strengths of the present study include a large and comprehensively characterized study population, 15‐year follow‐up time and comprehensive data on possible covariates. We used validated methods to measure LTPA^22^ and AGEs.^42^ We also recognize several limitations that might have caused bias in the findings. The LTPA questionnaires are generally susceptible to under‐ and overreporting of PA. SAF, as a measure of AGEs, is applicable mainly for non‐pigmented skin types, which could limit the replication of our findings in adults of European descent in more diverse populations. Our aging cohort is also prone to survival bias, where healthier individuals are more likely to participate in the study with advancing age. Furthermore, participants' levels of AGEs at late midlife were unknown, and there were only a few observations of extreme levels of PA, rendering a source of uncertainty in interpreting the results.

Considering the current data, the present study suggests that women who lead a sedentary lifestyle in late midlife have a less healthy aging trajectory by means of higher levels of AGEs in old age, compared with those who are more physically active. The results are interesting considering that most chronic diseases are age‐related, globally life expectancy increases, and physical activity is a readily available and reliable measure of improving health span.^43^ Future research would benefit from using objective data on PA, further investigating possible sex differences and working toward a unified measure of the aging process.

In conclusion, the volume of LTPA in late midlife is associated with AGEs in old age in women. We found a rapid decrease in levels of AGEs between 0 and 32 METh/week, where after the effect plateaus and loses significance. No conclusions about causality or directionality can be made from the present study. Our findings support the ongoing work to promote physical activity in older age.

## Disclosure statement

The authors declare no conflict of interest. The funding agencies had no influence on data gathering, analyses, interpretation of the data or writing of the manuscript.

## Supporting information


**Data S1.** Supporting Information.

## Data Availability

The data that support the findings of this study are available from the corresponding author upon reasonable request.
