# Supplementary material for: Leisure‐time physical activity predicts levels of advanced glycation end‐products in older women: A 15‐year follow up from the Helsinki Birth Cohort Study
Source: Geriatr Gerontol Int. 2025 May 1;25(6):781–8. doi: 10.1111/ggi.70049 (PMC12170969; doi:10.1111/ggi.70049)

**Leisure-time physical activity predicts levels of advanced glycation end products in older women – a 15-year follow-up from the Helsinki Birth Cohort Study**

**Supplementary file**

**Supplementary table 1**: Characteristics of men from the Helsinki Birth Cohort Study in 2001-2004 (n=339). Page 2-4.

**Supplementary table 2**: Characteristics of women from the Helsinki Birth Cohort study in 2001-2004 (n=428). Page 5-7.

**Supplementary table 3**: Linear regressions for AGEs in old age and covariates in late midlife for men (n=339). Page 8.

**Supplementary table 4**: Linear regression for AGEs in old age and covariates in late midlife in women (n=428). Page 9.

**Supplementary figure 1**: Minimally adjusted association between the volume of LTPA quartiles in late midlife and AGEs in men and women in old age. Page 10.

**Supplementary figure 2**: Minimally adjusted predictions and marginal effect of restricted cubic spline regression models between the LTPA in late midlife and AGE in men and women in old age. Page 11.

**Supplementary figure 3**: Flowchart of study participants in Helsinki Birth Cohort Study. Page 12.

**Supplementary table 1**

Characteristics of men from the Helsinki Birth Cohort Study in 2001-2004 (n=339)

|  |  | **LTPA I** | | | **LTPA II** | | | **LTPA III** | | | **LTPA IV** | | |  |
| --- | --- | --- | --- | --- | --- | --- | --- | --- | --- | --- | --- | --- | --- | --- |
| **Variable** |  | **n** | **mean (SD)** | | **n** | **mean (SD)** | | **n** | **mean (SD)** | | **n** | **mean (SD)** | | **p for trend** |
| Age, years |  | 82 | 60.3 | (2.3) | 85 | 61.3 | (2.8) | 76 | 61 | (2.4) | 96 | 61.8 | (2.6) | 0.0005 |
| Body mass index, kg/m2 |  | 82 | 27.6 | (4.2) | 85 | 26.7 | (3.1) | 76 | 27.1 | (3.2) | 96 | 27.1 | (3.8) | 0.53 |
| Triglycerides, mmol/l |  | 82 | 1.59 | (0.87) | 85 | 1.54 | (0.83) | 76 | 1.37 | (0.74) | 96 | 1.4 | (0.73) | 0.048 |
| Total cholesterol, mmol/l |  | 82 | 5.9 | (1.01) | 85 | 5.69 | (0.9) | 76 | 5.72 | (1.02) | 96 | 5.9 | (1.01) | 0.81 |
| HDL-cholesterol, mmol/l |  | 82 | 1.46 | (0.38) | 85 | 1.43 | (0.35) | 76 | 1.48 | (0.38) | 96 | 1.54 | (0.36) | 0.07 |
| LDL-cholesterol, mmol/l |  | 79 | 3.71 | (0.81) | 84 | 3.6 | (0.76) | 75 | 3.61 | (0.83) | 94 | 3.71 | (0.84) | 0.9 |
| Systolic BP, mmHg |  | 82 | 144.8 | (17.9) | 85 | 142.2 | (18.1) | 76 | 144 | (18.2) | 96 | 144.8 | (17.8) | 0.81 |
| Diastolic BP, mmHg |  | 82 | 91.4 | (9.8) | 85 | 88.4 | (9) | 76 | 88.9 | (10.9) | 96 | 89.7 | (9.4) | 0.36 |
| Dietary index, AU |  | 82 | 11.1 | (3.2) | 85 | 10.7 | (3.5) | 76 | 11.1 | (3.1) | 96 | 10.9 | (3) | 0.9 |
| Fasting glucose, mmol/l |  | 82 | 6.05 | (1.26) | 85 | 5.74 | (0.73) | 76 | 5.74 | (1.04) | 96 | 5.73 | (0.6) | 0.03 |
| 30 min glucose, mmol/l |  | 80 | 9.98 | (2.37) | 85 | 9.41 | (1.94) | 75 | 9.06 | (1.82) | 95 | 9.24 | (1.74) | 0.01 |
| 120 min glucose, mmol/l |  | 81 | 8.41 | (4.11) | 85 | 7.66 | (2.22) | 75 | 7.04 | (2.99) | 96 | 6.92 | (2.53) | 0.0006 |
| Fasting insulin, mU/l |  | 82 | 11.18 | (11.98) | 85 | 10.46 | (6.99) | 76 | 9.76 | (6.09) | 96 | 9.85 | (6.33) | 0.24 |
| 30 min insulin, mU/l |  | 80 | 63.6 | (42.34) | 85 | 70.15 | (50.38) | 75 | 70.01 | (41.45) | 96 | 69.46 | (43.9) | 0.44 |
| 120 insulin, mU/l |  | 81 | 74.93 | (55.31) | 85 | 84.16 | (86.42) | 75 | 62.02 | (52.49) | 96 | 63.28 | (49.85) | 0.06 |
| Maximum adulthood SES, n(%) |  |  |  |  |  |  |  |  |  |  |  |  |  | 0.33 |
| High official |  | 82 | 18 | (22) | 85 | 21 | (25) | 76 | 25 | (33) | 96 | 28 | (29) |  |
| Low official |  | 82 | 31 | (38) | 85 | 29 | (34) | 76 | 20 | (26) | 96 | 21 | (22) |  |
| Self-employed |  | 82 | 8 | (10) | 85 | 8 | (9) | 76 | 10 | (13) | 96 | 8 | (8) |  |
| Manual workers |  | 82 | 25 | (30) | 85 | 27 | (32) | 76 | 21 | (28) | 96 | 39 | (41) |  |
| Smoking, n(%) |  |  |  |  |  |  |  |  |  |  |  |  |  | 0.16 |
| Never |  | 82 | 29 | (35) | 85 | 28 | (33) | 76 | 26 | (34) | 96 | 21 | (22) |  |
| Quit |  | 82 | 36 | (44) | 85 | 45 | (53) | 76 | 35 | (46) | 96 | 61 | (64) |  |
| Current |  | 82 | 17 | (21) | 85 | 12 | (14) | 76 | 15 | (20) | 96 | 14 | (15) |  |
| Alcohol usage, n(%) |  |  |  |  |  |  |  |  |  |  |  |  |  | 0.88 |
| 3-7 times/week |  | 82 | 18 | (22) | 85 | 21 | (25) | 76 | 18 | (24) | 96 | 24 | (25) |  |
| 1-2 times/week |  | 82 | 45 | (55) | 85 | 39 | (46) | 76 | 35 | (46) | 96 | 41 | (43) |  |
| 1-2 times/month |  | 82 | 10 | (12) | 85 | 17 | (20) | 76 | 13 | (17) | 96 | 20 | (21) |  |
| ≤ Once a month |  | 82 | 9 | (11) | 85 | 8 | (9) | 76 | 10 | (13) | 96 | 11 | (11) |  |

BP=Blood pressure, HDL=High-density lipoprotein, LDL= Low-density lipoprotein, LTPA= Leisure-time physical activity, SES= Socioeconomic status, SD= standard deviation

**Supplementary table 2**

Characteristics of women from the Helsinki Birth Cohort study in 2001-2004 (n=428)

|  | **LTPA I** | | | **LTPA II** | | | **LTPA III** | | | **LTPA IV** | | |  |
| --- | --- | --- | --- | --- | --- | --- | --- | --- | --- | --- | --- | --- | --- |
| **Variable** | **n** | **mean (SD)** | | **n** | **mean (SD)** | | **n** | **mean (SD)** | | **n** | **mean (SD)** | | **p for trend** |
| Age, years | 111 | 61 | (2.9) | 108 | 60.6 | (2.5) | 115 | 61.3 | (2.8) | 94 | 61.7 | (3.1) | 0.04 |
| Body mass index, kg/m2 | 111 | 27.6 | (4.2) | 108 | 27 | (5) | 115 | 27 | (3.9) | 94 | 27 | (4.6) | 0.37 |
| Triglycerides, mmol/l | 111 | 1.44 | (0.81) | 108 | 1.32 | (0.74) | 115 | 1.48 | (0.8) | 94 | 1.43 | (0.76) | 0.69 |
| Total cholesterol, mmol/l | 111 | 5.86 | (1.03) | 108 | 6 | (1.08) | 115 | 6.1 | (1.07) | 94 | 6.14 | (1.04) | 0.04 |
| HDL-cholesterol, mmol/l | 111 | 1.68 | (0.39) | 108 | 1.79 | (0.43) | 115 | 1.74 | (0.44) | 94 | 1.79 | (0.44) | 0.14 |
| LDL-cholesterol, mmol/l | 108 | 3.56 | (0.87) | 105 | 3.59 | (0.88) | 114 | 3.7 | (0.96) | 92 | 3.7 | (0.9) | 0.19 |
| Systolic BP, mmHg | 111 | 143.5 | (20.3) | 108 | 140.3 | (18) | 115 | 141.7 | (20.4) | 94 | 144.2 | (22.8) | 0.76 |
| Diastolic BP, mmHg | 111 | 87.5 | (10.3) | 108 | 86.3 | (9.7) | 115 | 86.9 | (9.3) | 94 | 87.3 | (10.4) | 0.98 |
| Dietary index, AU | 111 | 10.3 | (3) | 108 | 11.5 | (3) | 115 | 10.8 | (3) | 94 | 10.6 | (2.9) | 0.65 |
| Fasting glucose, mmol/l | 111 | 5.48 | (0.94) | 108 | 5.6 | (1.11) | 115 | 5.42 | (0.87) | 94 | 5.42 | (0.75) | 0.41 |
| 30 min glucose, mmol/l | 108 | 8.92 | (1.98) | 108 | 9.1 | (2.22) | 115 | 8.67 | (1.95) | 94 | 8.85 | (1.91) | 0.45 |
| 120 min glucose, mmol/l | 108 | 7.42 | (2.86) | 108 | 7.29 | (2.63) | 115 | 7.22 | (2.48) | 93 | 7.1 | (2.49) | 0.37 |
| Fasting insulin, mU/l | 111 | 8.78 | (4.77) | 108 | 8.43 | (5.87) | 115 | 9.32 | (13.6) | 94 | 11.9 | (21.2) | 0.08 |
| 30 min insulin, mU/l | 108 | 69.78 | (38.62) | 108 | 61.7 | (39.76) | 115 | 76.18 | (45.48) | 94 | 70.56 | (51.34) | 0.36 |
| 120 insulin, mU/l | 109 | 80.84 | (54.14) | 107 | 66.7 | (52.21) | 115 | 65.6 | (38.61) | 94 | 75.09 | (72.57) | 0.38 |
| Maximum adulthood SES, n(%) |  |  |  |  |  |  |  |  |  |  |  |  | 0.53 |
| High official | 111 | 15 | (14) | 108 | 16 | (15) | 115 | 14 | (12) | 94 | 8 | (9) |  |
| Low official | 111 | 74 | (67) | 108 | 67 | (62) | 115 | 67 | (58) | 94 | 56 | (60) |  |
| Self-employed | 111 | 5 | (5) | 108 | 9 | (8) | 115 | 13 | (11) | 94 | 12 | (13) |  |
| Manual workers | 111 | 17 | (15) | 108 | 16 | (15) | 115 | 21 | (18) | 94 | 18 | (19) |  |
| Smoking, n(%) |  |  |  |  |  |  |  |  |  |  |  |  | 0.14 |
| Never | 111 | 72 | (65) | 108 | 70 | (65) | 115 | 66 | (57) | 94 | 49 | (52) |  |
| Quit | 111 | 18 | (16) | 108 | 23 | (21) | 115 | 33 | (29) | 94 | 30 | (32) |  |
| Current | 111 | 21 | (19) | 108 | 15 | (14) | 115 | 16 | (14) | 94 | 15 | (16) |  |
| Alcohol usage, n(%) |  |  |  |  |  |  |  |  |  |  |  |  | 0.88 |
| 3-7 times/week | 111 | 18 | (22) | 108 | 21 | (25) | 115 | 18 | (24) | 94 | 24 | (25) |  |
| 1-2 times/week | 111 | 45 | (55) | 108 | 39 | (46) | 115 | 35 | (46) | 94 | 41 | (43) |  |
| 1-2 times/month | 111 | 10 | (12) | 108 | 17 | (20) | 115 | 13 | (17) | 94 | 20 | (21) |  |
| ≤ Once a month | 111 | 9 | (11) | 108 | 8 | (9) | 115 | 10 | (13) | 94 | 11 | (11) |  |

BP=Blood pressure, HDL=High-density lipoprotein, LDL= Low-density lipoprotein, LTPA= Leisure-time physical activity, SES= Socioeconomic status, SD= standard deviation

**Supplementary table 3: Linear regression for AGEs in old age and covariates in late midlife in men (n=339)**

| Minimally adjusted | | | Fully adjusted | |
| --- | --- | --- | --- | --- |
| Independent variable | **Coefficient (95% CI)** | **p-value** | **Coefficient (95% CI)** | **p-value** |
| BMI | 0.021 (0.006-0.036) | 0.006 | 0.022 (0.007-0.037) | 0.003 |
| Dietary index | -0.001 (-0.018-0.016) | 0.899 | 0.004 (-0.013-0.021) | 0.635 |
| Triglycerides | 0.045 (-0.022-0.113) | 0.187 | 0.043 (-0.024-0.111) | 0.209 |
| Cholesterol | -0.045 (-0.099-0.009) | 0.1 | -0.041 (-0.095-0.013) | 0.133 |
| HDL | -0.15 (-0.293--0.006) | 0.041 | -0.122 (-0.266-0.022) | 0.096 |
| LDL | -0.06 (-0.127-0.006) | 0.076 | -0.062 (-0.129-0.005) | 0.07 |
| Systolic BP | 0.002 (-0.001-0.005) | 0.149 | 0.002 (-0.001-0.005) | 0.151 |
| Diastolic BP | 0.002 (-0.004-0.007) | 0.499 | 0.003 (-0.003-0.008) | 0.359 |
| Fasting glucose | 0.111 (0.054-0.167) | 0.0001 | 0.119 (0.063-0.176) | 0.00004 |
| 30 min glucose | 0.046 (0.02-0.072) | 0.001 | 0.048 (0.022-0.075) | 0.0004 |
| 120 min glucose | 0.022 (0.005-0.039) | 0.012 | 0.024 (0.007-0.041) | 0.007 |
| Fasting insulin | 0.012 (0.005-0.018) | 0.0004 | 0.012 (0.006-0.019) | 0.0002 |
| 30 min insulin | 0.001 (-0.0003-0.002) | 0.133 | 0.001 (-0.0002-0.002) | 0.095 |
| 120 min insulin | 0.0004 (-0.0005-0.001) | 0.386 | 0.001 (-0.0003-0.001) | 0.191 |

BMI = body mass index. HDL = high-density lipoprotein. LDL=low-density lipoprotein. BP=blood pressure

**Supplementary table 4: Linear regression for AGEs in old age and covariates in late midlife in women (n=428)**

| Minimally adjusted | | | Fully adjusted | |
| --- | --- | --- | --- | --- |
| Independent variable | **Coefficient (95% CI)** | **p-value** | **Coefficient (95% CI)** | **p-value** |
| BMI | 0.008 (-0.002-0.018) | 0.126 | 0.008 (-0.002-0.018) | 0.122 |
| Dietary index | -0.009 (-0.023-0.006) | 0.237 | -0.006 (-0.021-0.009) | 0.457 |
| Triglycerides | -0.002 (-0.058-0.055) | 0.955 | -0.019 (-0.077-0.039) | 0.519 |
| Cholesterol | -0.03 (-0.071-0.012) | 0.158 | -0.03 (-0.072-0.011) | 0.15 |
| HDL | -0.067 (-0.17-0.037) | 0.205 | -0.027 (-0.134-0.081) | 0.627 |
| LDL | -0.028 (-0.077-0.021) | 0.265 | -0.032 (-0.082-0.018) | 0.206 |
| Systolic BP | 0.001 (-0.002-0.003) | 0.606 | 0.001 (-0.001-0.003) | 0.372 |
| Diastolic BP | -0.001 (-0.006-0.003) | 0.631 | -0.0004 (-0.005-0.004) | 0.852 |
| Fasting glucose | 0.022 (-0.025-0.07) | 0.362 | 0.028 (-0.019-0.075) | 0.246 |
| 30 min glucose | -0.007 (-0.029-0.015) | 0.53 | -0.005 (-0.027-0.017) | 0.647 |
| 120 min glucose | 0.004 (-0.013-0.021) | 0.657 | 0.003 (-0.014-0.02) | 0.728 |
| Fasting insulin | -0.002 (-0.005-0.002) | 0.352 | -0.002 (-0.005-0.002) | 0.319 |
| 30 min insulin | -0.0001 (-0.001-0.001) | 0.881 | -0.0001 (-0.001-0.001) | 0.893 |
| 120 min insulin | -0.0001 (-0.001-0.001) | 0.863 | -0.0002 (-0.001-0.001) | 0.667 |

BMI = body mass index. HDL = high-density lipoprotein. LDL=low-density lipoprotein. BP=blood pressure

**Supplementary figure 1**

Minimally adjusted association between the volume of LTPA quartiles in late midlife and AGEs in men and women in old age.

AGE= Advanced glycation end-products. AU= Arbitrary units. LTPA= Leisure-time physical activity

**Supplementary figure 2**

Minimally adjusted predictions and marginal effect of restricted cubic spline regression models between the LTPA in late midlife and AGE in men and women in old age.


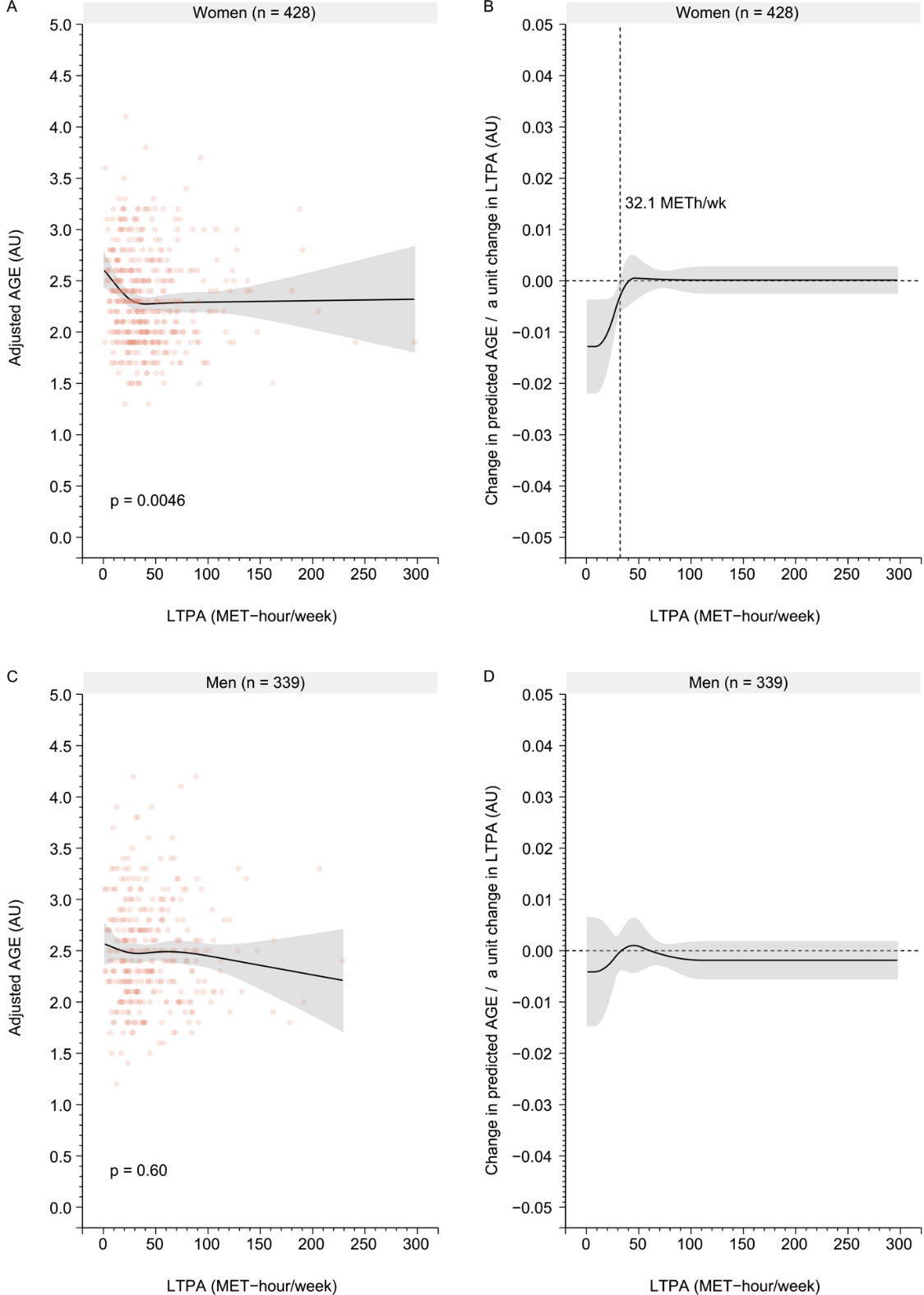


AGE= advanced glycation end-products. AU= Arbitrary units. BMI= body mass index LTPA= Leisure-time physical activity. MET= metabolic equivalent task

**Supplementary figure 3**

Flowchart of study participants in Helsinki Birth Cohort study.


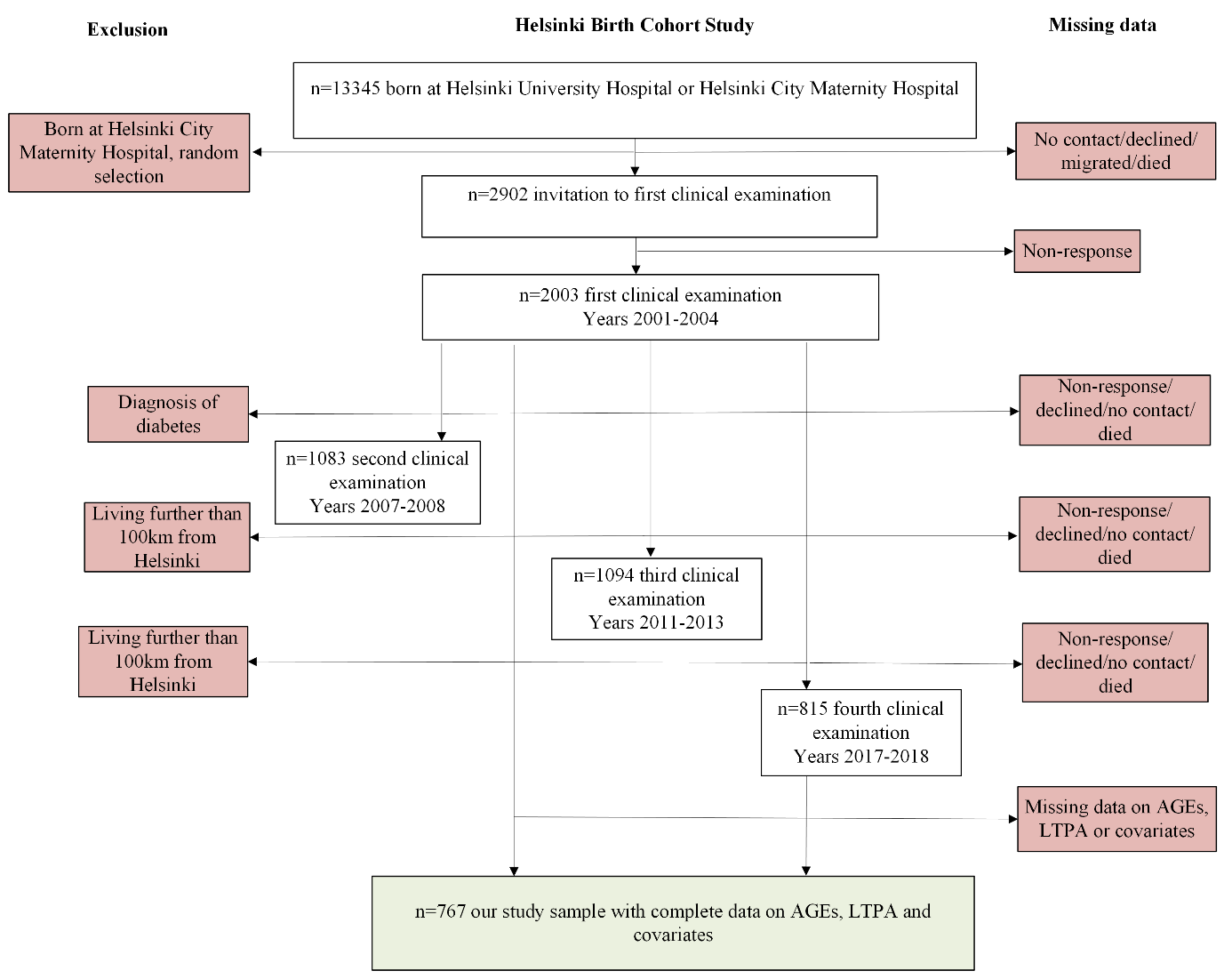

Supplement: Supplementary file 1 — Data S1. Supporting Information. [file GGI-25-781-s001.docx]
